# An interactive simulation and visualization tool for flood analysis usable for practitioners

**DOI:** 10.1007/s11027-015-9651-2

**Published:** 2015-05-29

**Authors:** Johannes G. Leskens, Christian Kehl, Tim Tutenel, Timothy Kol, Gerwin de Haan, Guus Stelling, Elmar Eisemann

**Affiliations:** 1 Water Engineering and Management, University of Twente, PO Box 217, 7500 AE Enschede, The Netherlands; 2 Computer Graphics & Visualization, Delft University of Technology, Mekelweg 4, 2628CD Delft, The Netherlands; 3 Computational Hydraulics, Stelling Hydraulics, Venezuelastraat 12, 2622 BR Delft, The Netherlands

**Keywords:** Flood, Visualization, Decision-making, Large-scale rendering

## Abstract

Developing strategies to mitigate or to adapt to the threats of floods is an important topic in the context of climate changes. Many of the world’s cities are endangered due to rising ocean levels and changing precipitation patterns. It is therefore crucial to develop analytical tools that allow us to evaluate the threats of floods and to investigate the influence of mitigation and adaptation measures, such as stronger dikes, adaptive spatial planning, and flood disaster plans. Up until the present, analytical tools have only been accessible to domain experts, as the involved simulation processes are complex and rely on computational and data-intensive models. Outputs of these analytical tools are presented to practitioners (i.e., policy analysts and political decision-makers) on maps or in graphical user interfaces. In practice, this output is only used in limited measure because practitioners often have different information requirements or do not trust the direct outcome. Nonetheless, literature indicates that a closer collaboration between domain experts and practitioners can ensure that the information requirements of practitioners are better aligned with the opportunities and limitations of analytical tools. The objective of our work is to present a step forward in the effort to make analytical tools in flood management accessible for practitioners to support this collaboration between domain experts and practitioners. Our system allows the user to interactively control the simulation process (addition of water sources or influence of rainfall), while a realistic visualization allows the user to mentally map the results onto the real world. We have developed several novel algorithms to present and interact with flood data. We explain the technologies, discuss their necessity alongside test cases, and introduce a user study to analyze the reactions of practitioners to our system. We conclude that, despite the complexity of flood simulation models and the size of the involved data sets, our system is accessible for practitioners of flood management so that they can carry out flood simulations together with domain experts in interactive work sessions. Therefore, this work has the potential to significantly change the decision-making process and may become an important asset in choosing sustainable flood mitigations and adaptation strategies.

## Introduction

Climate changes already have drastic implications for rainfall and ocean levels, and the situation is likely to worsen (Barros et al. 2014). Over 50 % of the world population lives in cities (WHO 2013) and more than two thirds of the largest cities are vulnerable to rising sea levels as a result of climate change (McGranahan et al. 2007). Hence, millions of people are therefore exposed to the risk of extreme floods and storms.

Developing strategies to mitigate or to adapt to the threats of floods is an important topic in the context of climate changes. Several countries and unions, including the European Union (EU), have put a multi-layer safety approach into place as a framework for the development of these mitigation and adaptation strategies (EU 2005). The multi-level safety approach consists of three layers. Layer 1 focuses on protection measures in the form of levees and dikes and has traditionally received most attention and funding (Kabat et al. 2009). For example, in the Netherlands, a 5-year maintenance cycle as well as a standardization for reinforcements has been established (STOWA 2008). Layer 2 consists of waterproof spatial planning, while layer 3 considers disaster management. Examples of mitigation measures through spatial planning (layer 2) are local protection of hospitals, schools, and utility companies or spatial planning measures that improve the chances of evacuation from a flooded area. Flood disaster management (layer 3) can be improved by setting up disaster protocols and practicing decision-making under worst-case scenarios.

One of the main challenges for following a multi-level safety approach is the integrated decision-making process it requires. This means that different practitioners, related to the three layers mentioned above, have to collaborate and find integrated solutions for flood management. These practitioners include policy analysts or political decision-makers from municipalities, water boards, and provinces or representatives from fire and police departments, hospitals, and energy companies.

In this process, it is important to build the capacity among these practitioners for involvement in flood management. Analytical tools, e.g., Sobek (http://www.deltaressystems.com/hydro/product/108282/sobek-suite) or Mike11 (http://www.mikebydhi.com), can be used to gain insight into the consequences of floods under different climate scenarios or alternative measures in terms of water depths, flow velocities, or damages. They offer predictions by simulating the physical processes involving various area features such as elevation and roughness resistance and external forces such as storm events and dam breaches (Al-Sabhan et al. 2003; Bates and Roo 2000; Moel and Aerts 2011; Stelling 2012a). To address model uncertainties, analytical tools are normally validated with historical events or when such measurements are not available, a sensitivity analysis or ensemble calculations can be carried out (Walker et al. 2003).

Up to the present, analytical tools have only been accessible to domain experts, such as hydraulic engineers and modelers, as the involved simulation processes are complex and rely on computational and data-intensive models. Practitioners (i.e., policy analysts and political decision-makers) are usually only presented with resulting maps. Typically, these indicate the impacts of individual flood hazards or the results of risk analyses, in which multiple hazard scenario’s, each having a certain likelihood, are combined into risk maps (Apel et al. 2004).

Despite the advantages that these maps may provide for practitioners of flood management, poor use is often made of this information for supporting decisions (Leskens et al. 2014b). Mors et al. (2005) show that practitioners of flood management, operating under regulatory, institutional, political, resource, and other constraints, prioritize other concerns over more sophisticated maps with model information about flood risks. An underlying reason might be the difference in perception of the threats of floods between domain experts, such as modelers or hydraulic engineers, and practitioners (Faulkner et al. 2007; Janssen et al. 2010; Timmerman et al. 2010; Wood et al. 2012). Domain experts generally frame flood issues using scientific knowledge and expertise. They assume that with more detailed model, information analyses will improve and better decisions can be made. Practitioners, on the other hand, often lack the capacity and time to incorporate the results of these complex analyses in their decisions.

A way to better support practitioners with the output of analytical tools is to improve their involvement in the application of these tools (Voinov and Bousquet 2010; Leskens et al. 2014b). This involvement can improve the alignment of the information requirements of practitioners with the opportunities and limitations of analytical tools (Leskens et al. 2014a). However, as mentioned, the existing analytical tools are only accessible to domain experts, as the involved simulation processes are complex and rely on computational and data-intensive models. These analytical tools have specialized interfaces and their application—for example, to picture a future flood scenario—takes multiple hours (Leskens et al. 2014b).

In this paper, we present a system that is focused on making the use of flood analysis tools accessible for practitioners of flood management, such that they can carry out flood analysis together with domain experts in interactive work sessions. The system gives the user the possibility to test several disaster scenarios and to receive direct visual feedback. It provides realistic images to help practitioners in interpreting these outputs of the system. We conducted a user study to test the effectiveness of this interface and its outputs. In order to investigate the applicability in practice, we illustrated the use of our system for real-world data in a case study for the area of West-Friesland, The Netherlands.

This work makes a two-fold contribution: 1) we present a working system and show the utility of our approach, and 2) we explain the technical contributions needed to achieve a working solution. The paper offers an overview of our system and its possibilities, focusing on interaction and visualization. We also give a description of the novel techniques that have been used. The results of two tests are presented.

## Our system

In this section, we explain how the system was developed and give an overview of the key features and their technical backgrounds. For more detailed descriptions, we provide references to other publications.

### Development of the system

The system presented here builds upon a prototype developed between 2011 and 2014, involving researchers and engineers from Delft University of Technology, Deltares, and Nelen & Schuurmans.

The key features of the system are based on user requirements that were derived from 13 semi-structured interviews among policy analysts of the regional water board Hoogheemraadschap van Delfland (The Netherlands), conducted in 2011 and reported in Leskens et al. (2014a). The interviews focused specifically on three questions. (1) What is your task or role in decision-making processes? (2) What information do you require to carry out this task? (3) What functions do you need from an analysis tool that can be operated during a work session? From these 13 interviews, it emerged that the following capabilities were considered important for using an analysis tool during a work session with domain experts: (1) technical reliability, (2) the possibility to assess the effectiveness of multiple scenarios within the time horizon of a work session, and (3) understandable output for non-water specialists.

Based on these user requirements, the key features of our system focus on a realistic visualization of floods and interactivity to enable practitioners to explore various options for flood mitigation and adaptation measures rapidly, together with domain experts. To ensure technical reliability (i.e., the first user requirement), our system combines a high spatial resolution (i.e., 0.5 m by 0.5 m) and the inclusion of all relevant processes (i.e., overland flow, groundwater flow, canal flow, and sewer flow) (Stelling 2012b).

There are other flood analysis tools available that provide comparable features and user interfaces (Nóbrega et al. 2008; Bates and De Roo 2000). However, our system is unique in providing the distinct features in the required scale (>1000 km ^2^) and resolution (>10 height values per square meter). Making an interactive system with a realistic visualization for such large areas is a very challenging goal.

### Overview

#### Three dimensional (3D) visualization

Our 3D visualization is aimed at supporting practitioners in interpreting and understanding the impact of simulated flood hazards. It shows the properties of simulation results in three spatial dimensions and a realistic rendering, mapped to real-world phenomena. In respect to two dimensional (2D) visualizations, in which the impacts of flood hazards can only be viewed directly from above, the 3D visualization shows the impacts also from the side. Flood depths can therefore be examined without the use of a legend in which typically different shadings of blue are used to indicate the depth of a flood. The basis for this realistic 3D visualization is data from Light detection and range (LiDAR) scans coupled to output of water simulations. LiDAR data is collected by a technology that measures distance by illuminating a target with a laser and analyzing the reflected light. The result of these LiDAR scans, usually carried out by helicopters, are points specified in three spatial dimensions (*x*, *y*, *z*), with a resolution of around 15–50 points per square meter. These points, colored via aerial images, ensure recognition of the area and its features (e.g., houses, cars, and trees) in high detail. Water properties, based on the simulations, are projected realistically into this 3D visualization. For example, flow directions are visualized by moving waves and water depth is displayed by adjusting the light extinction. Furthermore, the system supports stereo rendering to create the illusion of depth, if the corresponding equipment is available. Extensive interactive device support gives users the possibility to navigate the 3D world with ease. This also allows us to adapt the system’s navigation to the cognitive and motoric capacities of different audiences (e.g., general public, museum visitors, decision-makers, managers, hydrologists, etc.). Our system builds upon an efficient out-of-core rendering system that displays large-scale light detection and ranging (LiDAR) data (Haan 2009, 2010; Kehl and de Haan 2012). Even though large-scale point-based rendering is a classic rendering topic (Dachsbacher et al. 2003; Rusinkiewicz and Levoy 2000), a solution that combines large-scale water and terrain visualizations was previously unavailable.

#### Interaction

**Fig. 1:**
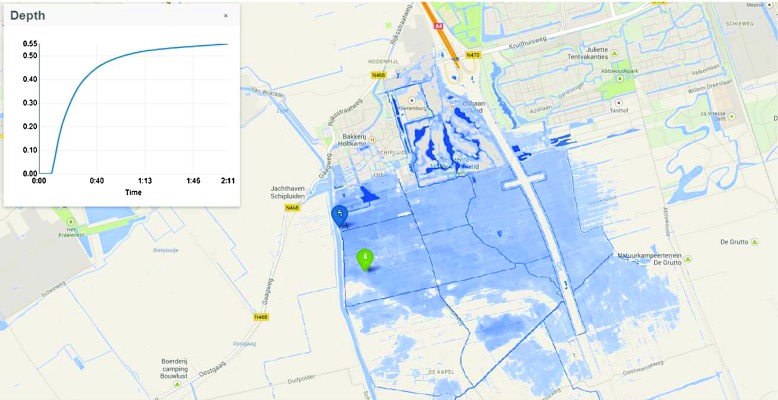
A screenshot of the 2D interface used for interaction

For interaction with the simulation process, our system relies on a simulation display that shows the map of the study area (Fig. 1). Different from the 3D visualization system, as presented in the former section, the study area in the simulation display can only be viewed directly from above. Once a simulation is carried out, the outcome can be presented in the 3D visualization system and be viewed by a helicopter perspective.

A user can choose two different methods to add water flows to the study area. First, a point source can be added, consisting of a flow discharge or a water level. This point source can, for example, be used to simulate a dam breach or a leaking sewer pipe. Second, a spatially distributed source can be added, consisting of a flow discharge to a selected area, which can be used to simulate rainfall events. Several properties can be selected for both types of water sources, for example, whether the flow discharge of a point source is constant or time-dependent, or whether the distributed discharge has a circular or an irregular shape. The impact of these sources on the area can be assessed simultaneously with the simulation process. The water depths are indicated by a dynamic blue overlay and can also be assessed by a graph on random points or over a random cross section. Flow velocity is indicated by bubbles in the water.

Aside from the various options to add water sources, the properties of the study area’s terrain can be adjusted as well. This can be done in three ways. First, the elevation of the area can be locally increased or decreased—for example, to simulate the effectiveness of digging passages in local elevations that obstruct water flowing to storage areas or to test the effectiveness of elevating levees with sand bags. Second, the land use type can be changed—for example, to increase the rainwater infiltration capacity of the ground by changing paved areas into unpaved areas. Third, the water system in the study area, consisting of canals, pumps, weirs, and culverts, can be adjusted—for example, to test the effectiveness of stronger pumps, broader canals, or higher weirs.

Adding water sources to the study area and adjusting the study area itself can be done at any time by the user during the simulation process. The simulation can also be stopped to make adjustments to the water sources or the study area, after which the process is resumed. The display is made accessible via an internet browser. Therefore, users do not need to install special software on their computers. In case multiple users access the system at the same time, one of the users has the authority to make adjustments to the simulation. This depends on the pre-specified user rights.

The interactive character of the simulation display, as described above, is made possible by a strong computation core. This computation core consists of numerical methods to solve the 2D hydraulic equations for water flow, under the conditions of short computation times while still conserving the details of the study area. The technical details of this flood simulation model can be found in Stelling (2012b) and Casulli and Stelling (2013).

### Technical contributions

Several hurdles were overcome to obtain a system that is accessible to practitioners who are no domain experts. We added four extensions to the existing 3D visualization techniques (Haan 2010; Kehl and de Haan 2012; Kehl et al. 2013). First, we introduce a solution to directly manipulate the LiDAR point sets to improve collaborative aspects. Second, we present a new visualization technique to add rainfall information. Third, we developed a scalable solution for water rendering to make it possible to investigate large-scale flooding scenarios. Finally, our system can be easily executed on network-connected display devices, hereby abstracting the used hardware—whether it be a single screen, a multi-screen setup, or a stereo device. In the following, we will give a short overview of these technical contributions.

#### Direct interaction with data points

To facilitate discussions and support multi-user interaction, we designed a web-based solution for *collaborative* interaction that allows concurrent users to highlight areas or even modify data. The aforementioned changes in the simulation display (terrain lifting, soil changes, etc.) and annotations can be transferred directly to the virtual 3D LiDAR model. The on-the-spot visualization of concurrent changes on 3D topographic models of comparably sized datasets has not been demonstrated before within the scientific geospatial community. Our solution also allows highlighting of areas via common user interfaces, such as Lizard, Google Maps, or Open Street Maps to ensure a good acceptance of the system among the various users. Such possibilities can be helpful for discussions by directly visualizing the impact of the suggested solutions (Isenberg et al. 2013). The input applications can be executed on widely used smart devices (e.g., tablets and smartphones), making our algorithms accessible to a wide audience.

The modifications can manipulate any point property, such as color, but also height. The algorithm reads as an input keyhole markup language (KML) files, which are readily produced with the abovementioned software packages. These 2D polygonal definitions are shared among various users, who can then concurrently define modifications.

Technically, the approach modifies the LiDAR points on the fly by restricting the changes to the points that are currently visible to the observer in the 3D visualization software. Hereby, we avoid treating the entire data set, which would be too large to allow for interactive rates. Each LiDAR point is localized on the fly via a hierarchical structure build from the user-defined polygonal areas in the KML file (Kehl et al. 2013). The latter stores in each area the wanted attribute modification. This modification can then be applied to the localized LiDAR point, prior to being drawn on the screen.

#### Rainfall visualization

Previous 3D visualization systems produced realistic imagery from simulation data, which improves understanding for practitioners. In this work, we further introduce a rainfall data visualization, which is particularly interesting for urban floods; dense construction (e.g., buildings, pavements, roads) and their disadvantageous infiltration properties lead to water accumulating on the pavement.

**Fig. 2:**
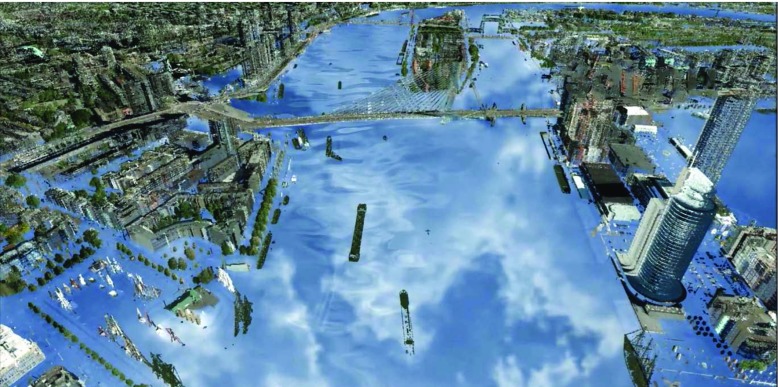
Cloud reflection for a flood in the city of Rotterdam due to a hypothetical scenario of heavy rainfall (100 mm/h)

In order to naturally embed precipitation information in a realistic 3D visualization, we developed a method to render, dynamically update and animate clouds according to measurements provided by the Koninklijk Nederlands Meteorologisch Instituut (KNMI). Figure 2 shows an example of the reflection of these clouds for an urban flood scenario in the city of Rotterdam, but the cloud layer is also directly visible, e.g., in a top-down view. We render satellite imagery (Holleman et al. 2009) as a realistic cloud layer, positioned 10 km above the ground, that is streamed in real-time into our 3D visualization software.

#### Water rendering

The rendering of large-scale water bodies is particularly challenging. No previous solution existed to directly render the results of hierarchical simulation processes (Stelling 2012b), despite the fact that such scenarios are very common in the investigation of large-area impacts.

The challenge is that the simulation relies on an approximation in the form of an adaptive quadtree, which respects water properties. These data sets, even in a hierarchical representation, are too large to be displayed entirely at real-time rates. Hence, an efficient display method is needed to simplify the representation. This adaptive scheme should be based on the viewing distance to add details only in the proximity to the observer, where they are needed.

The problem relates to terrain-rendering solutions, but previous work used a uniformly sampled height field (Losasso and Hoppe 2004; Baboud et al. 2011), while we need support for the hierarchical simulation results. Additionally, we want to support wave patterns to illustrate the underlying water properties, but previous work synthesized waves independently of any underlying simulation data (Ren and Zhou 2012) or did not allow for view-dependent simplifications (Kryachko 2004). These aspects are crucial for our large-scale data sets.

In our approach, we load and display only the simulation results that are currently in view. We use a hierarchical grid attached to the camera location that is very close to the observer and coarser in distance. As such, the grid resolution respects the distance to the observer. To transform this grid into a water representation, its vertices recover information from the underlying hierarchical flood simulation by querying the hierarchical structure based on their position. Depending on the stored water properties (presence, height, flow, and velocity), the vertices are displaced to represent the water levels and the recovered values are interpolated across each grid cell, which enables us to apply a texture-based wave-synthesis algorithm (van Hoesel 2011).

#### Display setups

We designed a display algorithm that distributes the workload among network-interconnected rendering machines. Such a solution makes it easy to run our solution on various 2D displays, multi-display setups, as well as 2D and 3D stereo projector systems. This is an interesting aspect as different visualization setups are common (Marton et al. 2012; Reda et al. 2013; Kuchera-Morin et al. 2014).

**Fig. 3:**
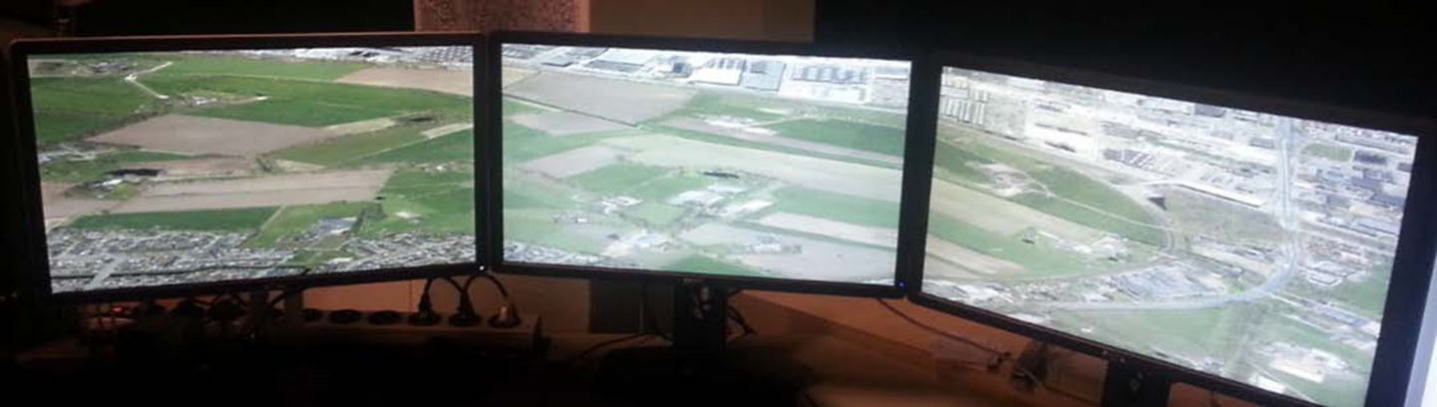
Multi-screen setup, visualizing the city of Delft on a three-screen panorama

Since particularly high-resolution results involve extensive computations in order to maintain interactivity at a high quality, our approach allows the involvement of multiple computers, which all contribute to the final images by sharing their results over the network. To illustrate the generality of this solution, we tested several different screen setups (screen walls of various screen arrangements, stereo devices, and cylindrical projection, as shown in Fig. 3). We also refer the interested readers to the accompanying video.

## Flooding analysis

To test whether the two key features of our system (i.e., realistic visualization and interactivity) can contribute to a better accessibility of flood analysis tools for practitioners and support collaborative flood analyses with domain experts, we carried out two tests. The first was aimed at the usability of our system for individual practitioners who are no domain experts. The second was a use case to test whether our system could support collaborative flood analysis with practitioners and domain experts in a real-world application.

We gathered the data input for our system from the aerial LiDAR scan AHN2 (Actueel Hoogtebestand Nederland - http://www.ahn.nl) together with satellite imagery. The data was further augmented by object databases and soil maps provided by the Dutch Water Boards and conversion tables to transform land use into infiltration rates, as well as roughness, interception, permeability, and porosity values (Cultuurtechnische Vereniging 1998). In common modeling practices, preferably a calibration and validation with real measured data is applied to assure the validity of the model outcomes. However, our case study was focused on testing the interface and user interaction and therefore a validation and calibration with real measured data was not necessary, although in this test, the outcomes had to be within a reasonable range of likelihood to be of use to the users.

### User study

In this part, we evaluate the usefulness of our system for practitioners of flood management, who are usually no domain experts. To this end, we asked the participants to perform an analysis themselves and to comment on different types of visualizations obtained by our system.

#### Participants

Seven subjects participated in our study (5 males and 2 females, aged between 24 and 46—the mean age was 30). All had normal or corrected-to-normal vision. While they were not familiar with the system and goals, they all had experience in working with computers.

#### Methodology

**Fig. 4:**
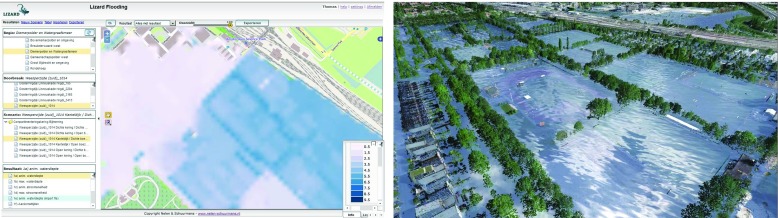
A simulated flood hazard in the Watergraafsmeer area represented with an illustration directly from above (2D) and a helicopter view (3D).

The experiment took around 15 min per participant, including instructions, test, and subjective feedback. It consisted of two parts: (1) a comparison between a simulated flood hazard represented with a illustration directly from above (2D) and a helicopter view (3D) (Fig. 4) and (2) an assignment in which the interactive simulation display was used.

For part 1 of the experiment, a flood was simulated in a study area in Amsterdam called Watergraafsmeer. The calculated maximum water depths of this simulation where projected in two ways. First, our 3D visualization system was applied, in which the user could make a realistic helicopter flight over the flooded area. Second, a conventional 2D visualization was applied, consisting of a digital map (Google), in which the maximum water depths where projected that could only be viewed directly from above (see Fig. 4). We showed both visualizations to each participant individually and asked them to respond to three closed questions and one open question:

1. Which visualization is best suitable to estimate damages to houses? [options: 2D, 3D, neutral]
2. Which visualization is best suitable to estimate loss of lives? [options: 2D, 3D, neutral]
3. Which visualization is best suitable to estimate whether evacuation is necessary? [options: 2D, 3D, neutral]
4. Does the 3D visualization have added value in respect to the 2D visualization and, and if so, what is this added value? [open question]

In part 2 of the experiment, the participants were asked to use the interaction possibilities to study the area Watergraafsmeer in Amsterdam. First, an introduction to our system was given to explain how the simulation display works. Second, the participants were given the opportunity to familiarize themselves with the simulation display. No explicit time limit was given for this. When they indicated that they understood how the simulation display had to be operated, they were asked to give an answer to the following question: which streets in the study area will be inaccessible for cars after a rain shower of 100 mm in 1 h? The time it took to answer this question was then measured.

#### Apparatus

We used an Intel Core i7 at 2.67 GHz processor, 6 GB of main memory, and an NVIDIA Quadro FX380 card with a 20-in. Samsung 2233RZ (120 H, 1680 × 1050 pixels) screen, and the participants used a classic three-button mouse device for interaction. For the 3D visualization, the main device uses an Intel Xeon 6 core hyperthreaded processor @ 3.2 GHz, 12 GB of main memory, and an NVIDIA GeForce GTX 680 with a common screen (60 Hz, 1920 × 1080 pixels) attached.

#### Results user study

The responses to the closed question in part 1 of the test (i.e., the comparison between a 2D and 3D visualization of the same flooded area) are listed in the table below (Table 1)

**Table 1:** The table presents the questions that where asked to each individual participant and, per question, the number of participants preferring a 2D/3D visualization

Question	2D Visualization	3D Visualization	Neutral
Which visualization is best suitable	2	5	0
for the estimation of damages to			
houses?			
Which visualization is best suitable	2	5	0
for the estimation of loss of lives?			
Which visualization is best suitable	3	3	1
for the estimation whether evacuation is necessary?			

The open question about what the added value of the 3D visualization was with respect to the 2D visualization was answered with the following statements:

- It makes it better possible to imagine the consequences of the flood.
- It enhances prediction of what a flood means for an area and helps to better empathize with the situation.
- It is more realistic and detailed. It is easier to interpret what a flood means for the area.
- It is more vivid and therefore better understandable.
- Less interpretation is required to estimate the consequences of the flood.
- It helps the user to better imagine how serious the flood is.
- It shows the consequences for the environment better.

The average time the participants needed to answer which roads would not be accessible for cars after a rain event of 100 mm was 6 min, with a minimum of 4 and a maximum of 7.

### Case study

To test whether our system is accessible for both model experts and practitioners such that they can carry out flood analysis together in an interactive way, we organized a case study. This case study was organized together with the Province of North-Holland and the Waterboard Hollands Noorderkwartier in the Netherlands. Various stakeholders of the pilot area West-Friesland were invited to a workshop to discuss flood mitigation and adaptation measures within the multi-level safety approach.

The West-Friesland area is a large, flood prone area (781 km ^2^), located north of Amsterdam and lies approximately 3 m under sea level. It is protected by dikes from water in the IJsselmeer/Markermeer-lake. The area is inhabited by approximately 400,000 people.

#### Set-up case study

A broad workshop was organized to create a decision-making environment in which various stakeholders in the area were informed about the threats of floods and were involved in investigating mitigation and adaptation measures in spatial planning and flood disaster management. During the workshop, the stakeholders were separated into groups to answer the central questions: “Which measures can be taken?” and “How can these measures be implemented?”. Ideas of the stakeholders, resulting from their own backgrounds and perspectives on the threats of floods, could immediately be tested and discussed using our system. The system was also used to produce maps ahead of time. The results are the following four maps, each post-processed per flood scenario:

1. Flooded roads: highways, secondary, and urban roads
2. Flooded utility companies: divided into electricity, gas, and water
3. Flooded vulnerable objects; hospitals, day care centers, generated schools, and old age homes
4. Arrival times in order to provide information for planning evacuations.

**Fig. 5:**
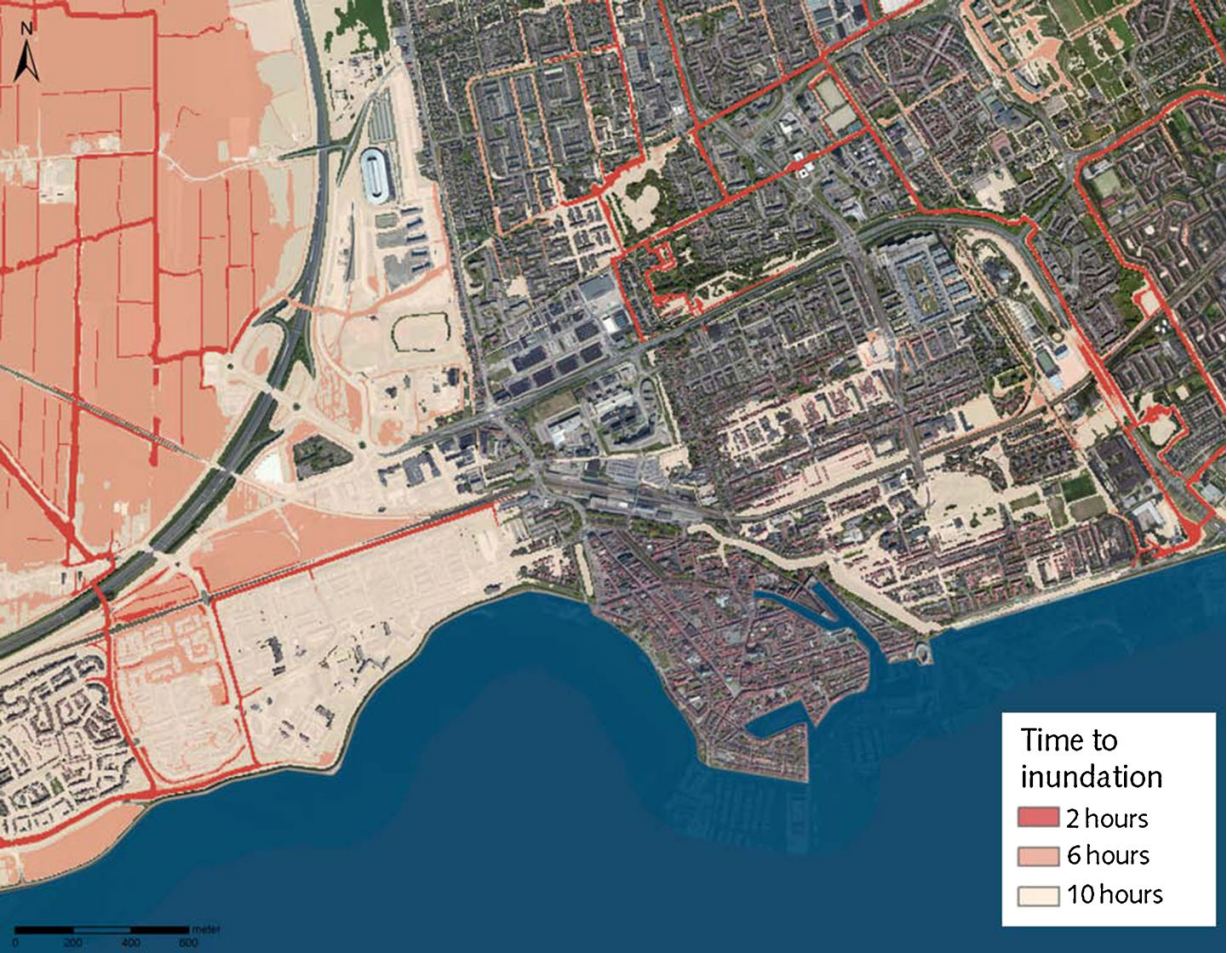
Arrival times—an exemplary map showing flood arrival times in different zones, which can be used for city evacuation planning

**Fig. 6:**
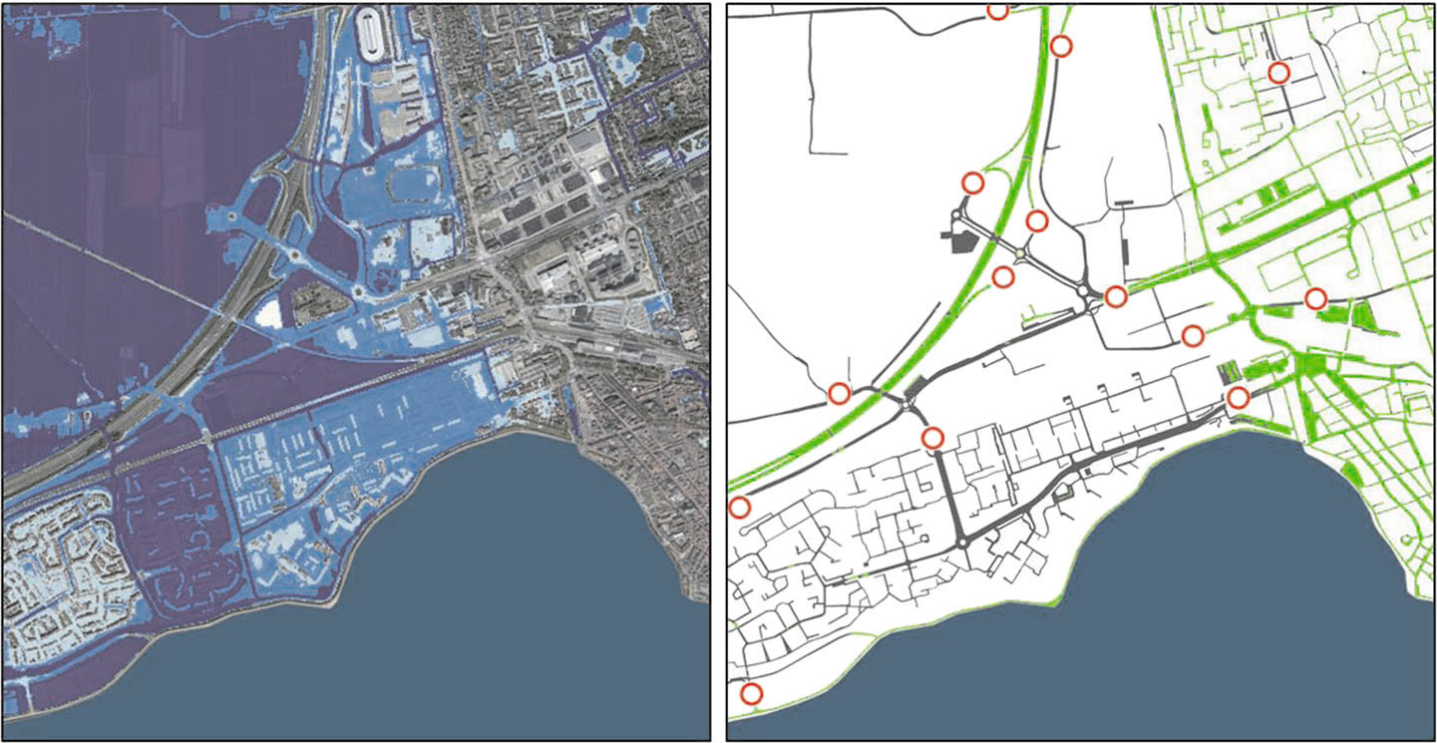
Accessibility of roads—another map resulting from the system that shows the mitigation of the flood (*left*) and the road access in the study area (*right*) accordingly. In the access map (*right*), *green paths* show usable road paths while *red-circled icons* symbolize access bottlenecks

Figure 5 shows an example of the arrival times of a simulated flood and Fig. 6 indicates the accessibility of roads.

#### Participants

Thirty-five stakeholders attended the workshop. This group existed of representatives of the province of North-Holland (official organizer of the workshop), the Waterboard, municipalities, agriculture, business, project developers, energy providers, health service, fire department, and an insurance company. These stakeholders covered most of the parties involved in choosing mitigation measures. Apart from five domain experts from a consultancy company and the water board who where used to applying analysis tools, most of the other participants were not used to involvement in the application of analysis tools.

#### Results case study

**Fig. 7:**
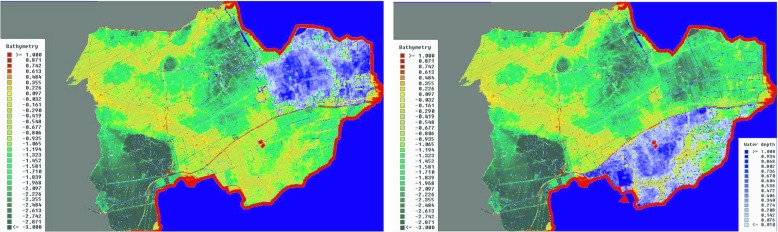
One investigated measure for flood protection is shown in this figure. The illustration shows the effect of dividing the area of West-Friesland in a southern part and a northern part by a dry dike (*red line*) at both dam breach locations

The work session yielded a number of measures that were proposed by the participants and could be directly tested with our system. The measures were assigned to responsible stakeholders for further elaboration. An overview of these measures is listed in Table 2. One of the measures that emerged during the workshop, which is considered to be of high potential, was dividing the area in different components by dry dikes (Fig. 7). The evaluation of the use of our system in the workshop indicated that a combination of interaction with the system and the realistic visualization allows the users to quickly judge the effectiveness of the solutions suggested in the various flood scenarios. The 3D visualization was considered a benefit over conventional 2D visualizations on maps. Users indicated that it was easier to estimate the impact of the calculated flood with the 3D visualization as they could relate the flood depths directly to familiar objects, such as trees, cars, and houses. With 2D visualizations, users first have to translate the different shades of blue into flood depths and then estimate the impact of the calculated flood. The positive impact on decision-making was attributed to the possibilities of testing the effectiveness of measures in a direct way. In the past, the effectiveness could only be evaluated by the intervention of model experts and was presented in a following work session. The post-processed map layers that illustrate additional information, such as damage, hindrance, and costs allocation, were deemed extremely useful for gaining additional insights beyond the physical values, such as water depths or flow velocity. Furthermore, this representation addressed the legal responsibilities of the different stakeholders.

**Table 2:** Flood mitigation and adaptation measures that were proposed by the participants of the work session and could be directly tested with our system

Stakeholder	Measure	Elaboration
Province	Include water safety in water	Use official strategic plan for
	regional planning	spatial planning and raise and
		maintain regional roads in order
		to provide evacuation routes
Municipality	Incorporate water safety in building	Adapt official building standards
	standards and regulations	
Water board	Create awareness and inform	Apply the water system to reduce
	stakeholders on water safety	the consequences of floods, for
		example, by using compartments
		in discharge canals
		Supply flooding data and
		information on a non-expert level
		Apply the water system for flood
		reduction and practice
Suppliers of energy	Ensure drinking water during	Keep pumping stations dry and
and water	flooding events, by keeping the	assure emergency power supply
	system under pressure (Electricity	Redirect mobile communication
	supply and communication	supply towards flooded area
	systems tend to break down easily)	
Companies and	Take private measures in case the	Take local measures such as dikes
entrepreneurs	level of protection ensured by the	around the property
	water board is not enough	
Inhabitants	Take private measures to survive	Prepare a survival kit
	for a longer period in case of	
	flooding	
Emergency services	Switch from procedural scripts to	Enhance evacuation scripts and
(fire departments,	scenario-related evacuations	the supply of information during
police)		calamities

The measures were assigned to responsible stakeholders for further elaboration

## Discussion

The results of our user study show that our system is accessible and usable for users who are not domain experts like hydraulic engineers or model specialists. First, the 3D visualization helped most of the participants to better understand the consequences of a flood scenario in terms of damages, loss of life, and the urgency to evacuate in respect to the 2D visualization. In the open question about the added value of the 3D visualization over the 2D visualization, all participants agreed that the 3D visualization helped them to better imagine what a flood means for an area. Second, it was shown that these non-expert participants all were able to use our interactive simulation display. Moreover, they were able to carry out an expert analysis about the availability of roads after a heavy rain event within minutes. With conventional models, which have specialist user interfaces and require hours of computation time, this would not have been possible.

The case study showed that our system was usable for collaborative application by practitioners and domain experts. The simulation display was effectively used on the level of at least three aspects. First, it enabled practitioners from different backgrounds to understand what floods meant for their specific field of profession. Second, it helped these practitioners to contribute in suggesting suitable solutions, following the multi-level safety approach. Third, the simulation display helped the practitioners to retrieve direct feedback on the effectiveness of their suggested solutions.

Our results show that our system is accessible for practitioners of flood management such that they can carry out flood analysis together with domain experts in interactive work sessions. However, one should be careful to draw general conclusions about the appreciations of our system. In our case, the simulation display and 3D visualization were mainly used for a general exploration of solutions which on the short term have no direct financial or political consequences. The impact of the decisions and the time pressure for making these decisions were therefore low. It is expected that our system will be used differently when time pressure and consequences are high. Experiences show that decision-makers in these circumstances highly value the accuracy of the outcomes of a model (Brugnach et al. 2007).

We expect that interactive modeling can contribute to a better understanding of uncertainties in model outputs among decision-makers as they can directly examine if current data were used in the model setup. They can also see how suggested solutions are translated into the model and therefore better understand the scope of the outcomes. Still, a calibration and validation with real measured data, as applied in common model practices, is always advisable. However, in environmental problems related to climate changes, the future is unpredictable and the nature of the relationship between processes is sometimes unknown (Leedal et al. 2010). In these cases, different model concepts or variation in input data in ensemble calculations can be considered (Renner et al. 2009; Walker et al. 2003). Methods to cope with model uncertainty, such as the use of real measured data or ensemble calculations, are all applicable with our system.

**Fig. 8:**
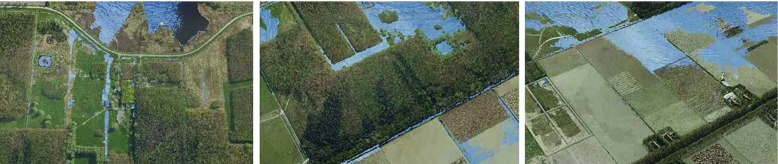
Our system is interactive and maintains a stable framerate. Here, we show an evolving flood during a real-time flythrough on a standard desktop computer

Regarding the technical aspects of our studies, we report that our system is efficient enough to be executed on a standard desktop PC, as reported in Section 3.1.3. For all scenarios, we could ensure a framerate of roughly 20 frames per second, which leads to a good tradeoff between speed and accuracy. Figure 8 shows a few images from a flythrough with an evolving flood. The interaction component was executed on a tablet but would run on most web-capable devices, which is a big advantage as it makes a large part of our system accessible anywhere and to anyone.

## Conclusions

In this paper, we presented our system for analyzing flooding scenarios. We showed that, despite the complexity of the involved models and the size of the involved data sets, our system is accessible for practitioners of flood management such that they can carry out flood analysis together with domain experts in interactive work sessions. In particular, the realistic 3D visualization helps practitioners, who are no domain experts, to better estimate the scale as well as the impact of a flood. The simulation display allows them to interact with the system and to explore complex flooding scenarios. Both results have been demonstrated in a user study and a case study, showing that our system represents an important step ahead towards the closer involvement of practitioners in the analysis driving the decision-making process for developing mitigation and adaption strategies for the threats of floods.

The accessibility of our system can also be underlined by the fact that a modified version was recently installed in two museums in The Netherlands, namely the Delft Science Center, as well as the Watersnoodmuseum in Zeeland. The two installations rely on our 3D visualization system to illustrate the impact of the 1953 flooding, which led to an enormous destruction of large parts of the Netherlands. Our solution gives people a direct access to flood-related information and offers insights into decision-making for flood mitigation and adaption strategies.
